# Resistance Temperature Detector for INHA RoSAT Rollable Solar Panel Using Electrohydrodynamic Inkjet Printing

**DOI:** 10.3390/s26196263

**Published:** 2026-10-02

**Authors:** Jin-Young Rim, Eo-Jin Jeon, Chang-Yull Lee

**Affiliations:** 1 Department of Aerospace Engineering and the Program in Aerospace Systems Convergence, Inha University, Incheon 21999, Republic of Korea; wgrzone01@inha.edu (J.-Y.R.); eojin@inha.edu (E.-J.J.); 2 Aerospace Systems Research Institute, Inha University, Incheon 21999, Republic of Korea

**Keywords:** solar panel, resistance temperature detector, electrohydrodynamic printing, polyimide coating, thermal cycling test

## Abstract

Rollable solar panels can increase the available photovoltaic area of CubeSats, but their flexible structure requires a flexible sensor for monitoring surface temperature during operation. In this study, an Ag-based flexible resistance temperature detector (RTD) was fabricated by electrohydrodynamic (EHD) inkjet printing for the rollable solar panel of the 3U CubeSat INHA RoSAT. The sensor was formed on a polyimide (PI) substrate and protected with a PI coating. Fourier transform infrared spectroscopy and thermogravimetric analysis were used to assess the imidization, residual solvent removal, and thermal stability of the coating. The sensor was then evaluated through an eight-cycle thermal cycling test from −25 to 125 °C, tailored with reference to MIL-STD-1540. The coating, heat-treated up to 250 °C, showed clear imide formation and no notable mass loss below 500 °C. During thermal cycling, the sensor exhibited stable resistance behavior with a drift rate of 0.032%/h. The derived calibration equation yielded a mean absolute error of 1.44 °C, while the temperature coefficient of resistance and hysteresis were 0.269%/°C and 1.2% of full-scale output (FSO), respectively. These results support the feasibility of the sensor for integration with rollable solar panels and future on-orbit temperature estimation.

## 1. Introduction

As the application scope of CubeSats has expanded and payloads have become increasingly sophisticated in recent years, securing a stable power supply within a limited shape and volume has emerged as a critical challenge in CubeSat design. In particular, a conventional body-mounted solar panel alone often cannot supply sufficient power to operate high-performance payloads or carry out complex missions [1,2]. Accordingly, extensive research has been conducted on deployable and flexible solar panels that can provide a larger photovoltaic area within a limited platform [3,4]. For example, Chamberlain et al. verified the on-orbit deployment and power generation performance of the Roll-Out Solar Array (ROSA), built on a flexible substrate, on the International Space Station [5]. Turse et al. developed the Composite Beam Roll-Out Array (COBRA), designed to generate approximately 200 W for a 6U satellite, and reported the results of vibration and zero-gravity deployment tests [6]. As such, flexible solar panels have been studied as a means of improving power generation capability under constrained stowage volume and mass.

However, the actual power output of a solar panel depends not only on its area but also on its operating temperature on-orbit. According to previous studies, the electrical output of a solar panel decreases by approximately 0.4–0.65% for every 1 °C increase in temperature [7]. This indicates that temperature variations directly affect power generation performance. In particular, because CubeSats must operate within a limited power budget, failure to account for the actual operating temperature of the solar panel makes it difficult to accurately estimate the available power margin. The surface temperature of the solar panel is therefore a key parameter for ensuring mission stability, and its on-orbit variation must be continuously measured [8]. Moreover, temperature measurement on structures subject to bending, such as flexible solar panels, requires a flexible sensor.

Printed electronics has been actively studied as a fabrication process for flexible sensors. It is a technology that fabricates electronic devices, such as sensors and displays, by printing conductive ink onto a substrate. Because conductive patterns can be formed directly on flexible substrates, this technology is well suited for fabricating flexible devices [9]. Among printed electronics techniques, electrohydrodynamic (EHD) inkjet printing is a non-contact printing method in which a fine ink jet is ejected by an electric field generated at the nozzle tip. Because the resulting jet diameter is smaller than the nozzle diameter and process parameters such as the applied voltage and flow rate can be precisely controlled, uniform and accurate patterns can be formed [9]. EHD inkjet printing has also been applied to the fabrication of temperature sensors. Ahn et al. fabricated a temperature sensor on a polyimide film using EHD printing with a Ag paste-based ink and evaluated its resistance characteristics [10]. Kamal et al. also fabricated a Ag nanoparticle-based temperature sensor on a polyethylene terephthalate (PET) substrate using EHD printing and evaluated its TCR, hysteresis, and repeatability [11]. However, few studies have applied an EHD-printed flexible temperature sensor to an actual satellite structure and verified its performance under representative space thermal conditions.

Applying a sensor to a spacecraft such as a satellite requires considering how the space environment, which differs from the terrestrial environment, affects the sensor. Space is a vacuum environment, and satellites in orbit are continuously exposed to large temperature variations as they repeatedly pass through sunlight and eclipse periods [12]. One risk factor that can arise in this environment is outgassing. Outgassing is a phenomenon in which residual solvents or volatile components inside a material are released under vacuum and high-temperature conditions. If the released substances adhere to the surface of a satellite’s sensors or optical systems, they can cause contamination and degrade system performance. To protect sensors from contamination and damage in such a space environment, a polyimide (PI) coating with excellent heat and chemical resistance can be applied [13]. As an example, Qian et al. applied a polyimide-based encapsulation film to protect flexible solar cells for space applications [14]. A PI coating, however, can act as a protective layer while also becoming a source of outgassing in the space environment. Windrich et al. reported that residual solvents inside a PI film can affect outgassing behavior and the thermal properties of PI and confirmed this through Fourier transform infrared (FT-IR) spectroscopy and thermogravimetric analysis (TGA) [15].

Repeated exposure to high- and low-temperature conditions in orbit can cause sensor damage or changes in electrical response. It is therefore necessary to verify whether the sensor maintains a stable response under such extreme temperature conditions. Thermal cycling testing is used as a method to simulate this repeated temperature environment and evaluate changes in sensor performance and potential damage [16]. Ji et al. explained that thermal cycling testing is widely used to demonstrate performance and evaluate the reliability of spacecraft electronics, and examined thermal cycling test conditions in terms of temperature range and cycle count based on the tailoring model presented in MIL-STD-1540 [17]. Accordingly, evaluating the feasibility of applying a sensor in space requires verifying that its performance and durability are maintained under repeated temperature variation.

In this study, an Ag-based flexible resistance temperature detector (RTD) was fabricated using EHD inkjet printing technology to measure the surface temperature of the rollable solar panel on the 3U CubeSat INHA RoSAT (Rollable Solar Array Testbed). The sensor was designed considering the shape of the rollable solar panel and the satellite’s temperature measurement system. A PI coating was also applied to protect the sensor, and the suitability of the coating for space application was evaluated through FT-IR and TGA. For the fabricated sensor, the test temperature range and number of cycles were tailored with reference to MIL-STD-1540, and a thermal cycling test was performed accordingly. The results of the thermal cycling test were used to analyze sensor stability, temperature–resistance characteristics, and hysteresis. The fabricated sensor was mounted on the rollable solar panel of INHA RoSAT and launched into space. However, on-orbit temperature data from the sensor have not yet been obtained, and efforts to communicate with the satellite and receive its data are still ongoing. Therefore, this paper focuses on the fabrication and performance evaluation of the sensor rather than on-orbit measurement results.

## 2. INHA RoSAT

### 2.1. Overview of INHA RoSAT Mission

The Department of Aerospace Engineering at Inha University developed INHA RoSAT, a 3U CubeSat independently designed and fabricated in-house. INHA RoSAT is a technology demonstration satellite that employs a rollable solar panel to mitigate the power generation limitations imposed by the restricted shape and volume of a CubeSat. The satellite was selected in the 2022 CubeSat competition organized by the Korea Aerospace Research Institute (KARI) and was launched into space aboard the fourth flight of the Nuri (KSLV-II) launch vehicle in November 2025. The rollable solar panel of INHA RoSAT is a flexible structure that remains stowed in a rolled state inside the satellite during ground storage and launch, and unfolds widely through a deployment mechanism once the satellite enters orbit and begins its mission. It therefore provides a larger area than a conventional body-mounted solar panel, which is constrained by the satellite’s shape. Through this design, the mission aims to relieve the power constraints imposed by the limited shape and volume of CubeSats and to increase the feasibility of operating high-power payloads in the future [3].

### 2.2. Temperature Sensor for Rollable Solar Panel

**Figure 1:**
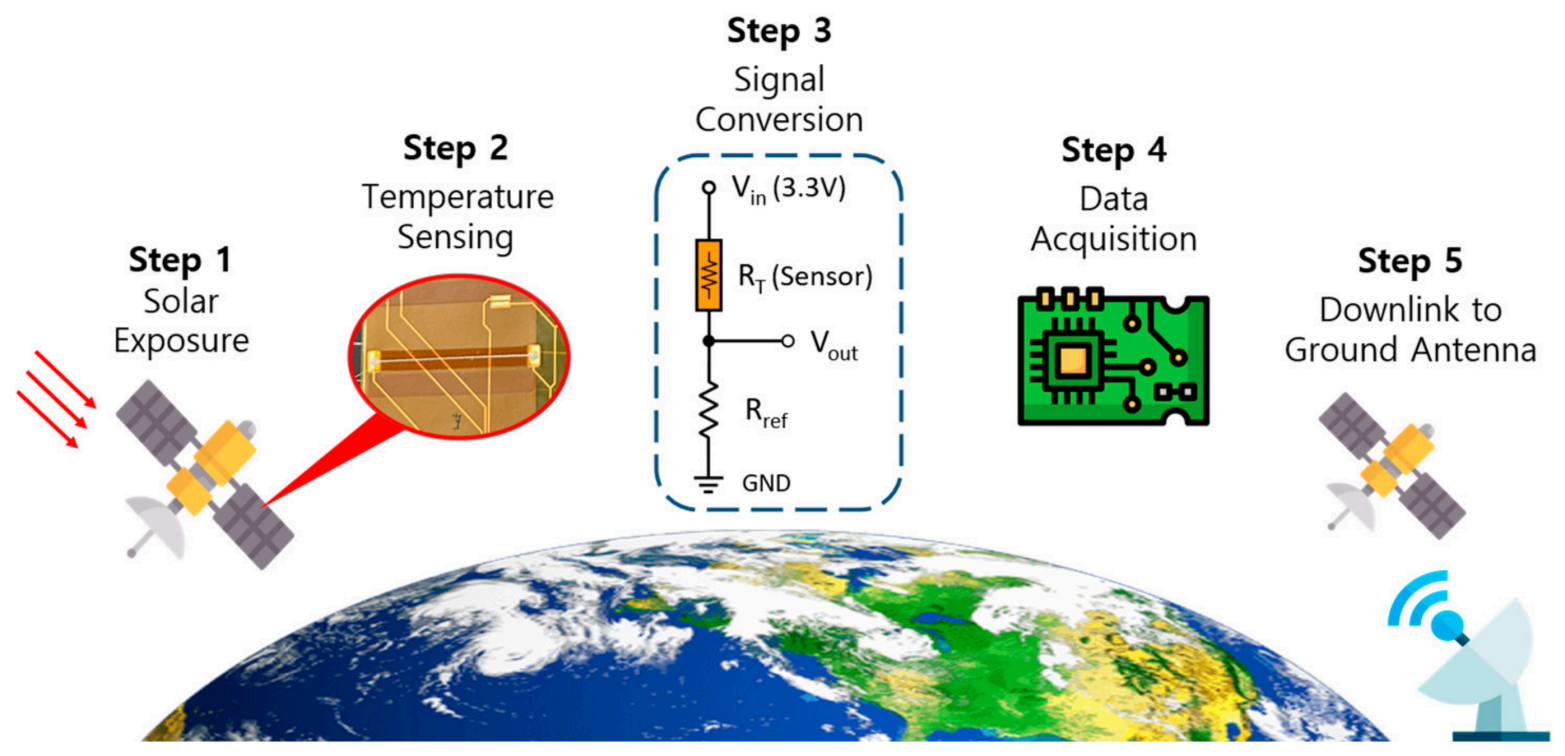
Data acquisition process for the temperature sensor in space.

**Figure 2:**
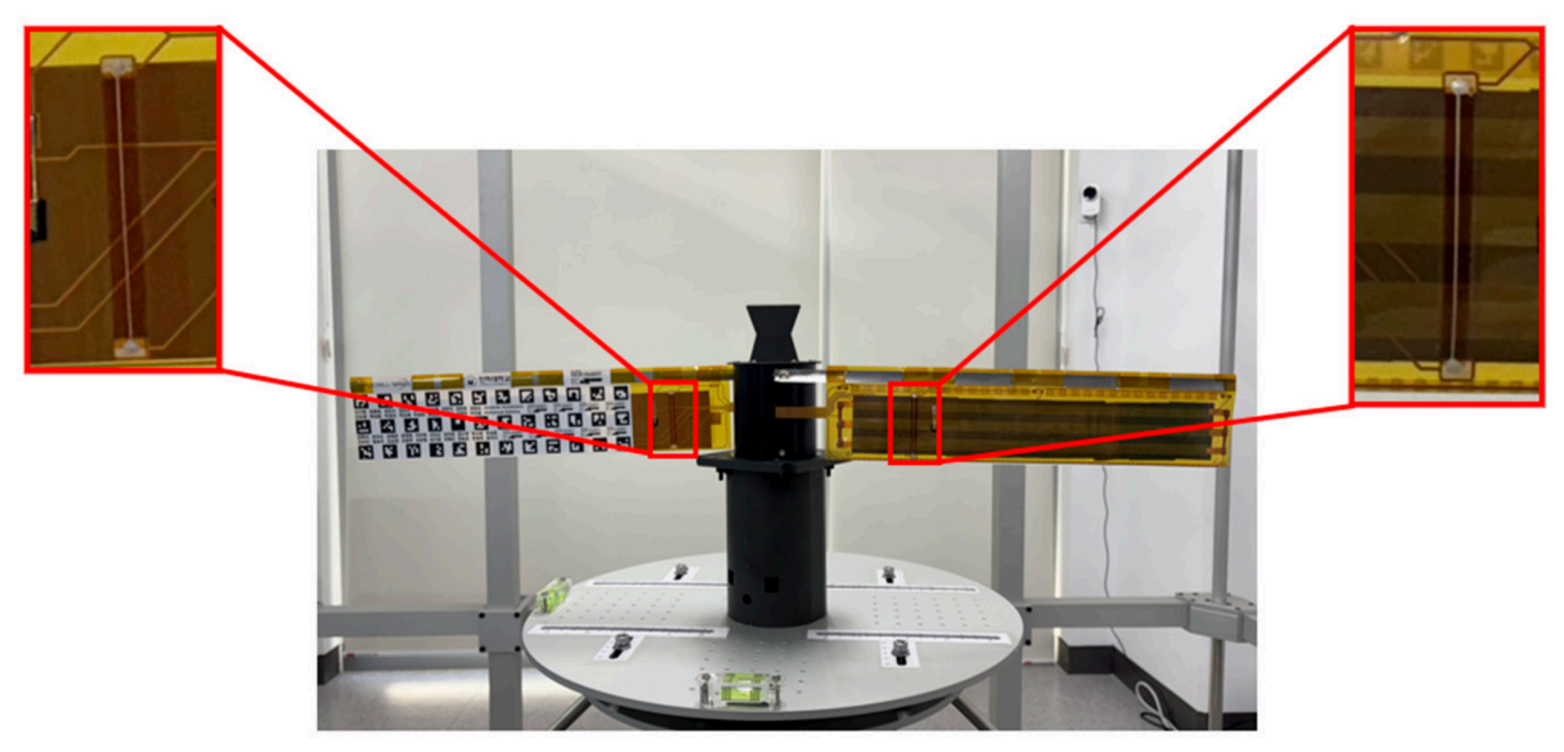
Temperature sensors applied to the rollable solar panel.

The flexible temperature sensor fabricated in this study was attached to the rollable solar panel to measure the panel’s temperature variation. Figure 1 illustrates the process of acquiring temperature sensor data in space, and Figure 2 shows the fabricated temperature sensor applied to the actual rollable solar panel of INHA RoSAT. The sensor operates on the RTD principle, and the sensor resistance *R*_T_ at temperature *T* can be expressed as in Equation (1) using the resistance *R*_0_ at the reference temperature *T*_0_ and the temperature coefficient of resistance *α*.

*R*_T_   =   *R*_0_[1   +   *α*(*T*  −  *T*_0_)]   (1)

The change in sensor resistance is converted into a voltage signal by a voltage divider circuit incorporating a 100 Ω reference resistor *R*_ref_ connected to GND. In this system, the sensor resistance *R*_T_ is connected on the input-voltage (*V*_in_) side and the reference resistance *R*_ref_ is connected on the GND side. The output voltage *V*_out_ of the voltage divider circuit can therefore be expressed as in Equation (2).

(2) $$V_{\mathrm{out}}\;=\;\;V_{\mathrm{in}}\frac{R_{\mathrm{ref}}}{R_{T}\;+\;\;R_{\mathrm{ref}}}\;\;$$

In this system, the input voltage *V*_in_ is set to 3.3 *V*. As the sensor temperature rises and *R*_T_ increases, the denominator increases, so the output voltage V_out_ of the voltage divider circuit decreases. In other words, in this circuit, a rise in the temperature of the solar panel appears as an increase in sensor resistance and a decrease in output voltage. The output voltage of the voltage divider circuit is delivered to the analog-to-digital converter (ADC) input of the in-house on-board computer (OBC) mounted on the satellite. The OBC converts the analog voltage signal into a digital value using a 16-bit ADC with a 3.3 *V* reference voltage and stores the converted data. The voltage resolution Δ*V* of the ADC is then given by Equation (3).

(3) $$\Delta V\;\;=\;\;\frac{V_{\mathrm{in}}}{2^{16}}\;\;=\;\;\frac{3.3}{65536}\;\;\approx \;\;50.35\;\;\mathrm{\mu V}$$

The data stored in the OBC are downlinked to the ground station via the satellite’s communication antenna once the satellite enters a communication window with the ground station. The ground station converts the received ADC digital output back into a voltage and calculates the sensor resistance using the voltage divider relationship. Rearranging the voltage divider equation in Equation (2) with respect to the sensor resistance *R*_T_ yields Equation (4).

(4) $$R_{T}\;\;=\;\;R_{\mathrm{ref}}\;\;\times \;\;\;\left(\frac{V_{\mathrm{in}}}{V_{\mathrm{out}}}\;\;-\;\;1\right)$$

Following the equations and data conversion process described above, the ADC digital data acquired by the satellite are sequentially converted into voltage and sensor resistance at the ground station. The calculated sensor resistance is then substituted into a previously obtained temperature–resistance relationship to estimate the temperature of the rollable solar panel.

## 3. Materials and Methods

### 3.1. Characterization of the Polyimide Coating

**Figure 3:**
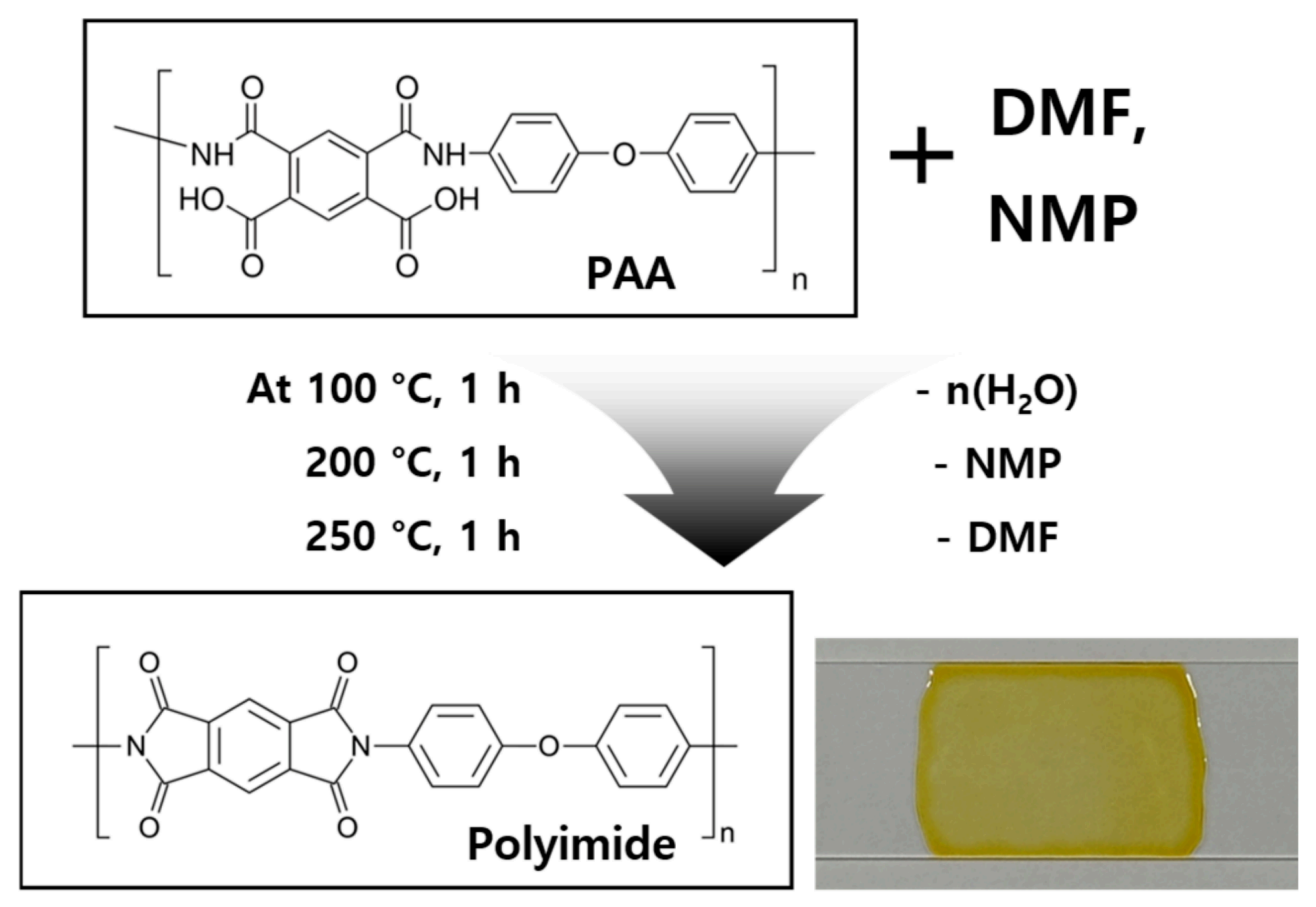
Flowchart of the PAA imidization process.

In this study, a PI coating was applied to protect the EHD-printed sensor from the space environment. Figure 3 illustrates the imidization process of the PI coating. The coating solution consists of a polyamic acid (PAA) precursor solution and N,N-Dimethylformamide (DMF). During heat treatment, the amic acid structure is converted into an imide ring structure. In this process, the solvents evaporate together with water, a byproduct of imidization, forming the PI coating layer. If the solvents do not evaporate sufficiently during heat treatment, outgassing may occur when the coating is exposed to the space environment. Therefore, FT-IR and TGA were performed to evaluate the degree of imidization and the presence of residual substances that could induce outgassing. FT-IR is an analytical technique that identifies the chemical functional groups of a material through its infrared absorption spectrum, allowing qualitative assessment of changes in specific chemical bonds. TGA is an analytical technique that measures changes in specimen mass as a function of temperature and can be used to assess the degree of residual solvent removal and the thermal stability of the material.

Specimens were prepared as free-standing films using a spin coater (SF-100A, Rhabdos, Seoul, Republic of Korea). The coating solution was prepared in the same manner as for subsequent sensor coating. To examine the degree of imidization at each stage, separate specimens were prepared under three heat-treatment conditions: 100 °C for 1 h, 100 °C for 1 h followed by 200 °C for 1 h, and 100 °C for 1 h followed by 200 °C for 1 h and 250 °C for 1 h. FT-IR measurements were performed using a VERTEX 80V (Bruker Optics GmbH & Co. KG, Ettlingen, Germany) in attenuated total reflectance (ATR) mode. The resolution was set to 2 cm^−1^, and spectra were measured over a wavenumber range of 600–4000 cm^−1^. To exclude the influence of differences in specimen thickness and contact with the ATR crystal, the data were normalized to the aromatic C=C stretching peak near 1500 cm^−1^. A TG 209 F3 (NETZSCH-Gerätebau GmbH, Selb, Germany) was used for TGA. Specimens were heated from room temperature to 800 °C at a rate of 10 °C/min under a nitrogen atmosphere, and mass change was measured with a resolution of 0.1 µg.

### 3.2. Fabrication Process

The sensor was fabricated using an EHD inkjet printing system (Enjet Inc., Suwon, Republic of Korea). The ink used for sensor fabrication consisted of Ag paste (Paron-910, Changsung Nanotech Co., Changwon, Republic of Korea) anddiethylene glycol monobutyl ether acetate (DGBA, Daejung Chemical & Metals Co., Siheung, Republic of Korea). The viscosity of the Ag paste is specified as 15–25 Pa·s at 50 rpm, while that of DGBA is approximately 5.6 mPa·s at 20 °C based on the product specifications. A PI film (Kapton^®^, DuPont, Wilmington, DE, USA) was used as the sensor substrate, and 3M 966 double-sided tape was used to attach the sensor to the solar panel. The PAA precursor used for the protective coating was poly(pyromellitic dianhydride-co-4,4′-oxydianiline) amic acid solution (15.0–16.0 wt% in N-methyl-2-pyrrolidone (NMP), Sigma-Aldrich, St. Louis, MO, USA), and DMF (Daejung Chemical & Metals Co., Siheung, Republic of Korea) was used as an additional solvent.

**Figure 4:**
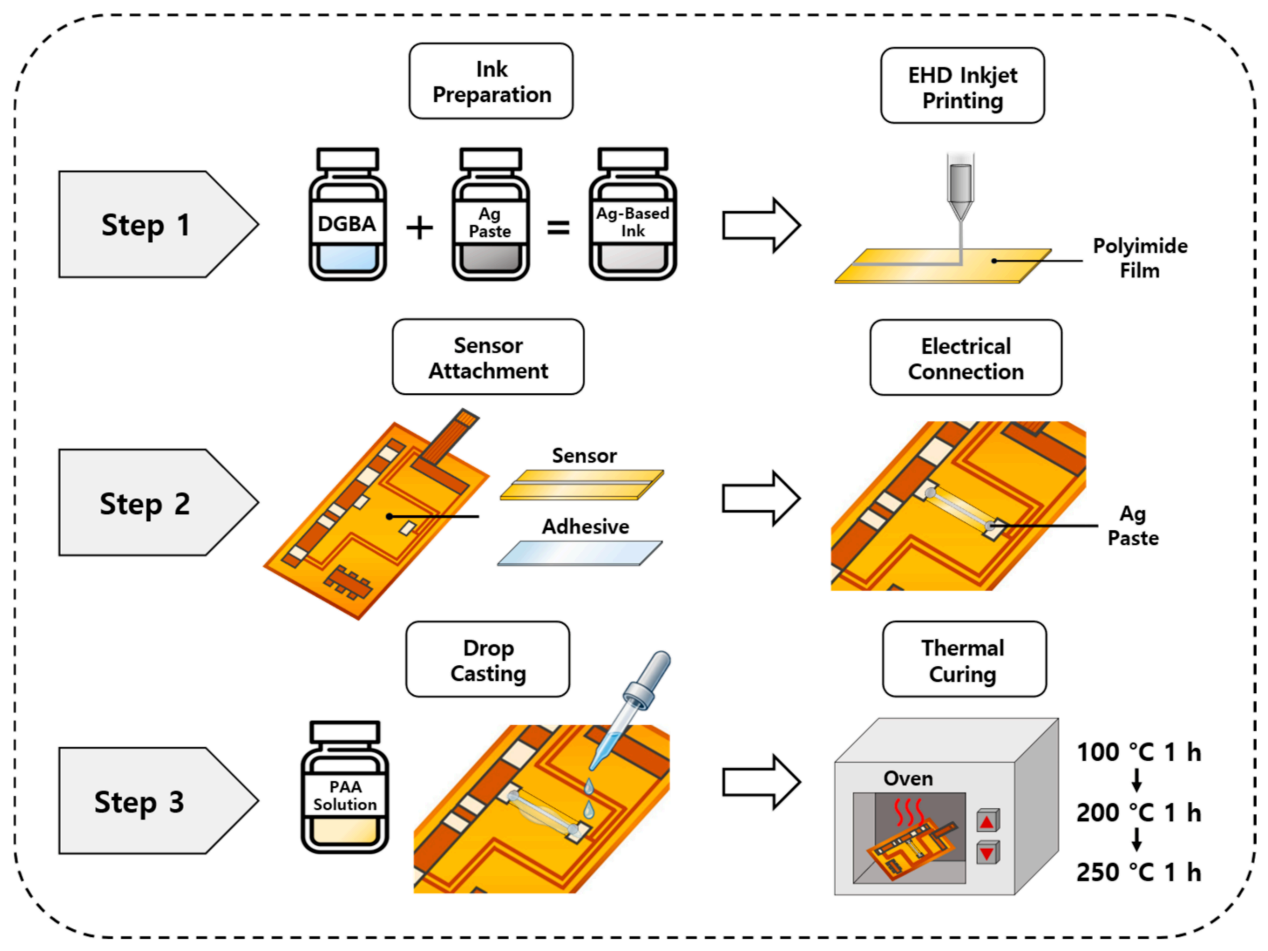
Schematic illustration of the fabrication process.

**Figure 5:**
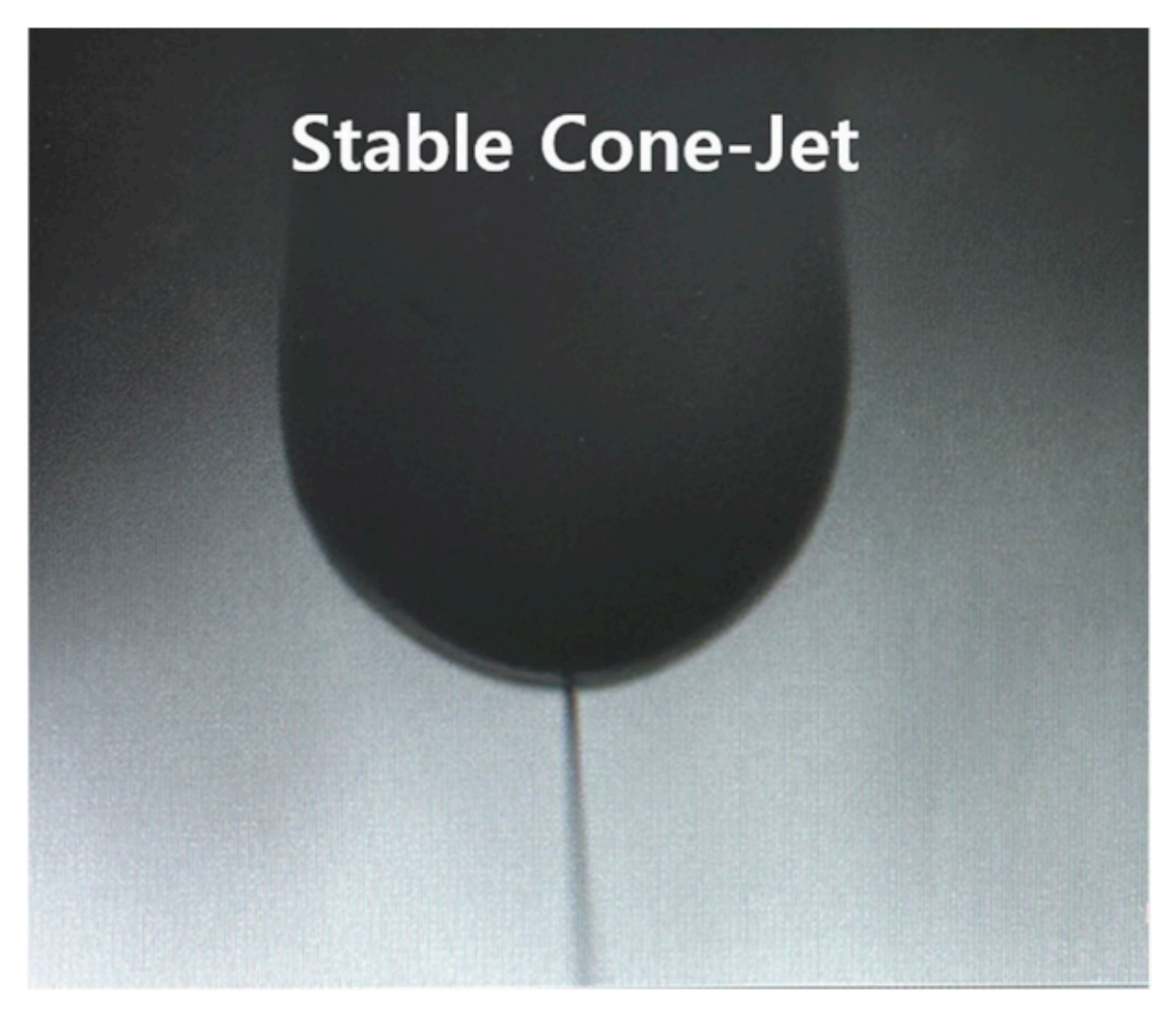
Stable cone-jet mode during EHD inkjet printing.

Figure 4 illustrates the sensor fabrication process. Figure 5 shows the stable cone-jet mode observed under the selected printing conditions, which are summarized in Table 1. The printing ink was prepared by mixing Ag paste and DGBA at a 1:1 mass ratio and stirring the mixture at 65 °C and 800 rpm for 24 h using a magnetic stirrer. In EHD inkjet printing, the ink is ejected most stably in the stable cone-jet mode, where the surface tension of the ink and the force generated by the electric field are in balance. Accordingly, the working position, applied voltage, and flow rate that maintained a stable cone-jet mode were selected as the printing conditions. The Ag pattern was formed using three printing passes under the same printing conditions.

**Figure 6:**
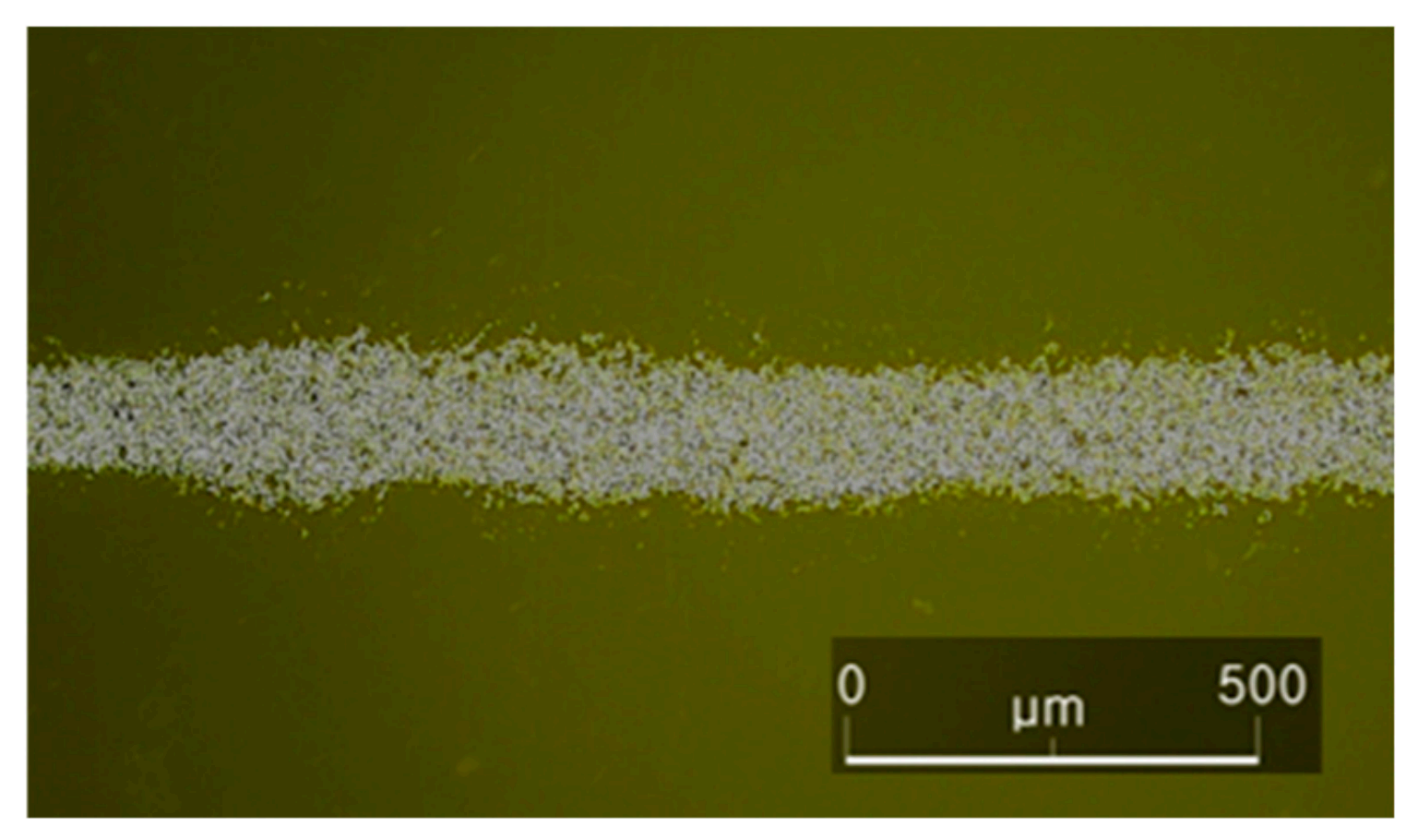
Optical micrograph of the printed Ag pattern.

**Figure 7:**
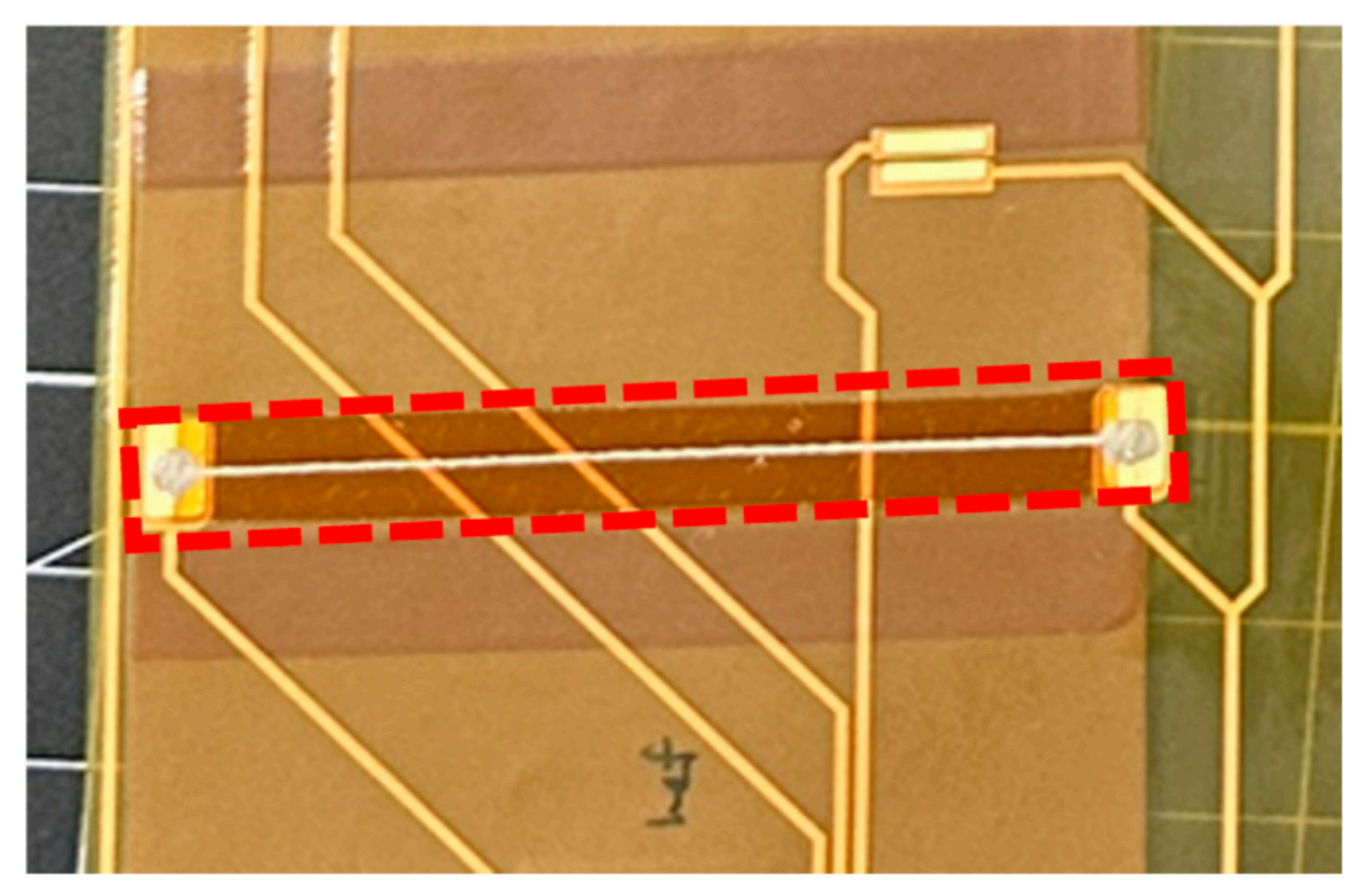
Photograph of the fabricated sensor.

Figure 6 shows an optical micrograph of the EHD-printed Ag pattern on the PI substrate, obtained using an optical microscope (ICS PRO-305B, Sometech Inc., Seoul, Republic of Korea) at 160× magnification. The printed Ag pattern exhibited a continuous line without apparent cracking or discontinuity. Figure 7 shows a photograph of the fabricated sensor, and Table 2 presents the specifications. The sensor was designed considering the characteristics of the voltage divider circuit. In a voltage-divider-type temperature measurement circuit, the output voltage range is determined by the ratio between the initial sensor resistance and the reference resistance. Accordingly, setting the initial sensor resistance to a level similar to the reference resistance is advantageous for stably measuring a wide temperature range within the ADC input range. In addition, if the initial sensor resistance is too low, the relative influence of contact resistance from the electrodes and wiring becomes more significant, potentially increasing temperature estimation error. Therefore, in this study, the target initial resistance of the sensor was set to approximately 100 Ω with reference to the resistance level of Pt100, a commercial RTD. The initial resistance was then examined as a function of line length, and an initial resistance close to 100 Ω was achieved at a line length of 50 mm. This printing condition was accordingly applied, and the reference resistance of the voltage divider circuit was also set to 100 Ω. 

The printed sensor was attached to the electrode area of the solar panel using 3M 966 tape, and Ag paste was applied to establish an electrical connection with the electrode. A protective coating was then applied to the sensor. The coating solution was prepared by mixing a PAA solution with DMF at a 1:1 mass ratio. The mixture was stirred at room temperature for 1 h using a magnetic stirrer and then sonicated for 30 min [18]. The prepared solution was applied by drop casting onto the sensor, including the electrode area. The coating layer was then formed by stepwise heating in an oven at 100 °C, 200 °C, and 250 °C for 1 h each.

### 3.3. Thermal Cycling Test

Thermal cycling tests simulate the repeated temperature variations to which satellites are exposed in space. These tests are used to verify component performance under such conditions and detect potential defects. However, the cost and time required for thermal cycling testing depend on the temperature range and number of cycles, which can greatly affect the development schedule and budget. Therefore, this study examined a tailoring process to widen the temperature range and reduce the number of cycles required for the thermal cycling test.

(5) $$N_{A}=10{\left(125/\Delta T_{A}\right)}^{1.4}$$

The tailoring equation for the acceptance-level thermal cycling test presented in MIL-STD-1540 is given in Equation (5). Here, *N*_A_ is the number of cycles determined by the temperature change range Δ*T*_A_. The number of cycles decreases as the temperature change range increases, and increases as the range decreases. To determine an appropriate *N*_A_, a margin was applied to the temperature range derived from this CubeSat’s operational orbit, setting the final test temperature range from −25 °C to 125 °C. Accordingly, with Δ*T*_A_ set to 150 °C, *N*_A_ was calculated to be 7.74. The untailored condition recommended in MIL-STD-1540 consists of 12.5 cycles, with a temperature range of 105 °C.

**Figure 8:**
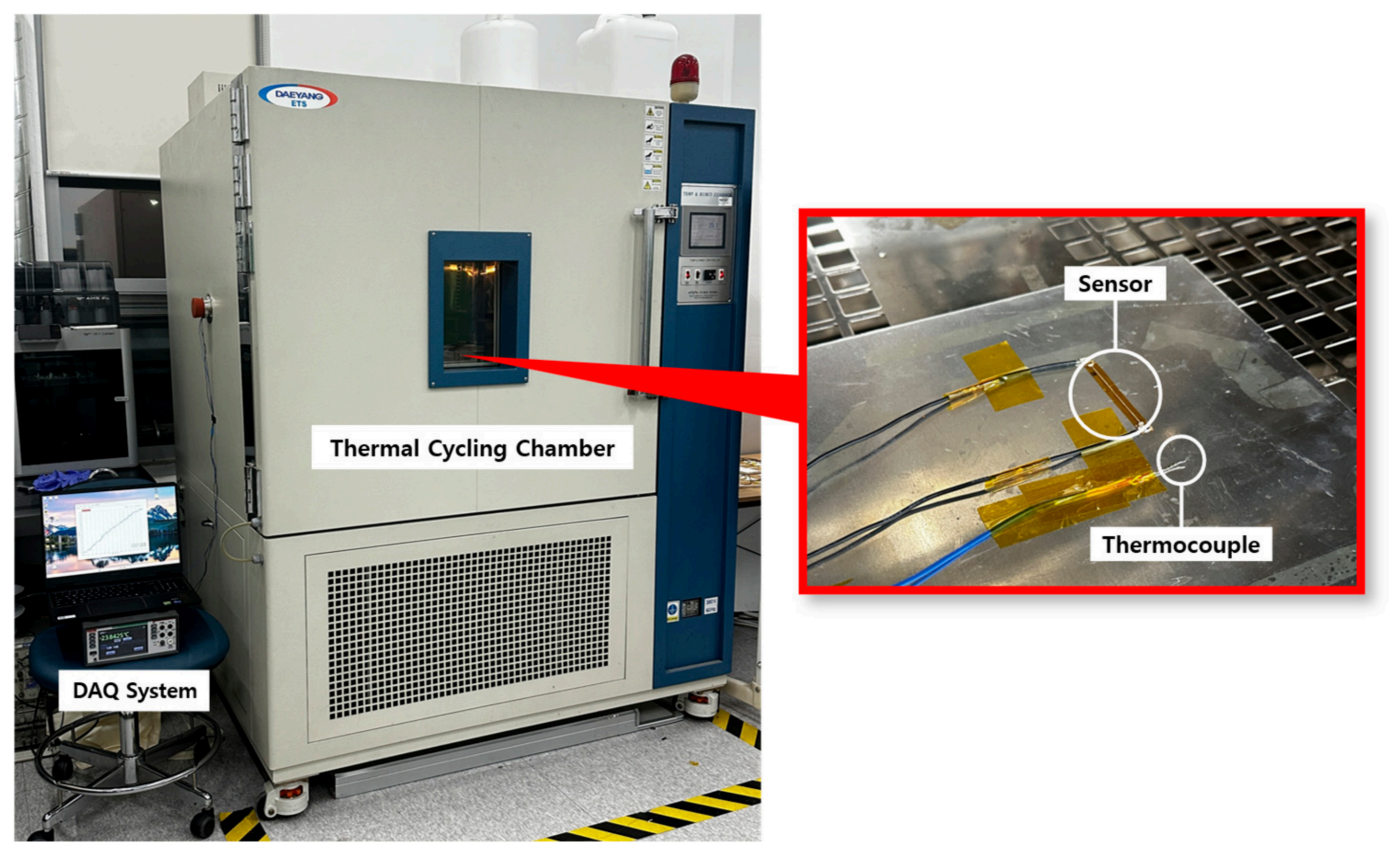
Experimental setup.

The final temperature profile selected for testing consisted of eight cycles. The test began and ended at room temperature, 25 °C, with a minimum temperature of −25 °C and a maximum of 125 °C. A dwell time of 6 h was applied at the minimum and maximum temperatures for the first and last cycles, while a dwell time of 1 h was applied for the intermediate cycles. The temperature ramp rate was set to be no slower than 1 °C/min. Figure 8 shows the experimental setup. The sensor and a thermocouple were fixed at adjacent positions inside a thermal cycling chamber (DYTH-252-P, Daeyang ETS, Pyeongtaek, Republic of Korea) so that both were subjected to the same temperature variation. This allowed the temperature measured by the thermocouple to be paired with the corresponding sensor resistance, enabling evaluation of sensor performance and derivation of the temperature–resistance relationship. Data were collected using a data acquisition system (DAQ6510, Keithley Instruments, Cleveland, OH, USA).

## 4. Results and Discussion

### 4.1. Assessment of the Polyimide Coating for Space Application

**Figure 9:**
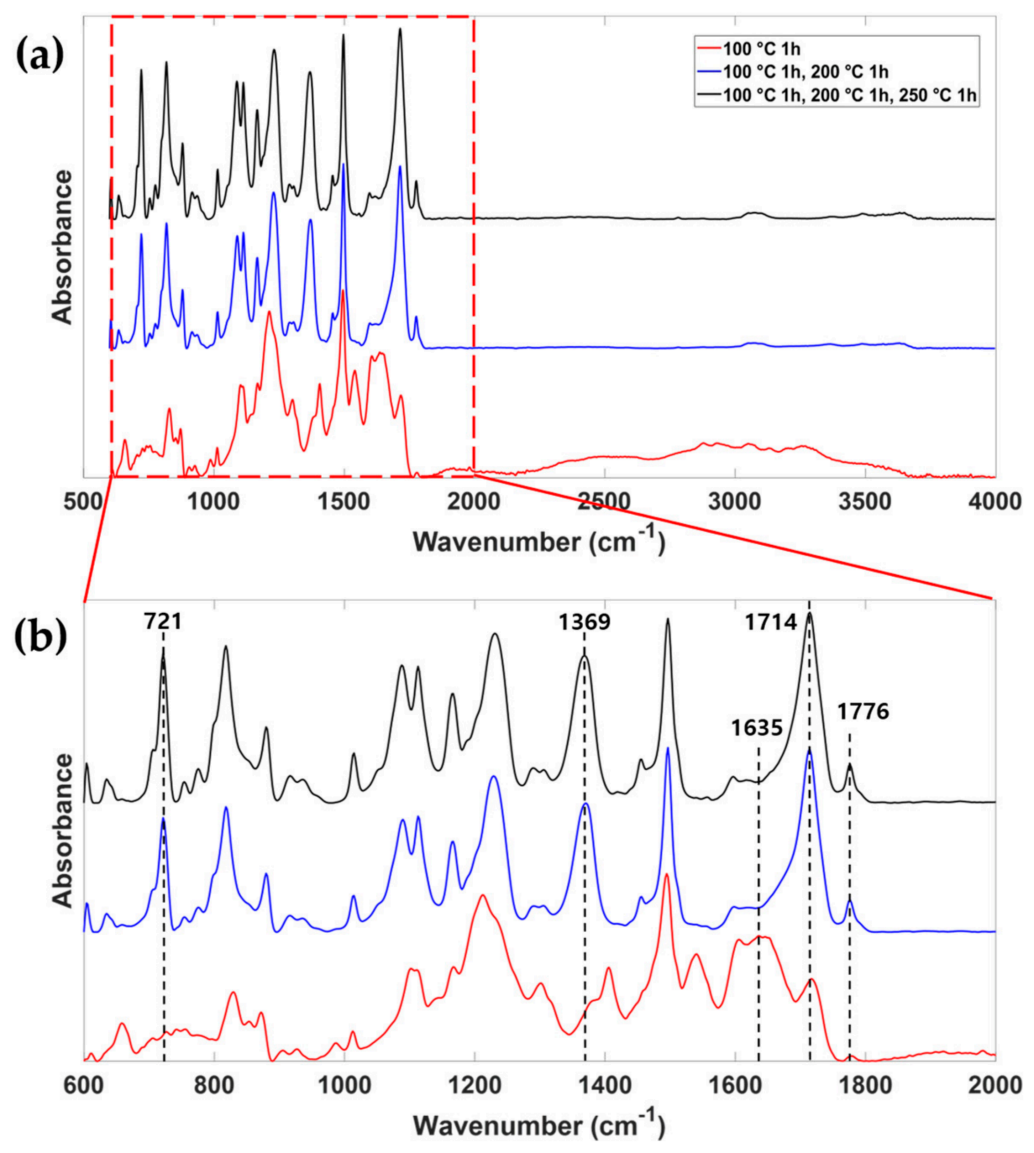
FT-IR spectra of the polyimide coating films under different heat-treatment conditions: (**a**) full spectra with the 600–2000 cm^−1^ region highlighted and (**b**) enlarged spectra in the 600–2000 cm^−1^ region.

Figure 9a shows the full FT-IR spectra of the PI coating films under stepwise heat treatment conditions. Figure 9b presents an enlarged view of the 600–2000 cm^−1^ region highlighted in Figure 9a for a clearer comparison of the imidization-related peaks. In the specimen heat-treated only at 100 °C, absorption near 1635 cm^−1^, corresponding to the amide C=O stretching of PAA, was clearly observed. In addition, the broad absorption observed in the 2500–3500 cm^−1^ region is attributed to incompletely imidized PAA and residual solvents. The broad O–H/N–H stretching associated with PAA appears mainly in the approximately 2500–3500 cm^−1^ region, while residual DMF and NMP contribute C–H stretching bands mainly around 2800–3000 cm^−1^. In the specimen heat-treated up to 200 °C, the PAA-related absorption near 1635 cm^−1^ decreased markedly, and characteristic absorption peaks of aromatic polyimide were clearly observed near 721, 1369, 1714, and 1776 cm^−1^. In the specimen heat-treated up to 250 °C, these characteristic peaks appeared even more distinctly. These results confirm that, as the heat treatment temperature increased, the amic acid structure of PAA was progressively converted into the imide structure. Therefore, the stepwise heat treatment applied up to 250 °C effectively induced imidization of the PAA precursor, confirming that it is an appropriate heat treatment condition for the PI coating.

**Figure 10:**
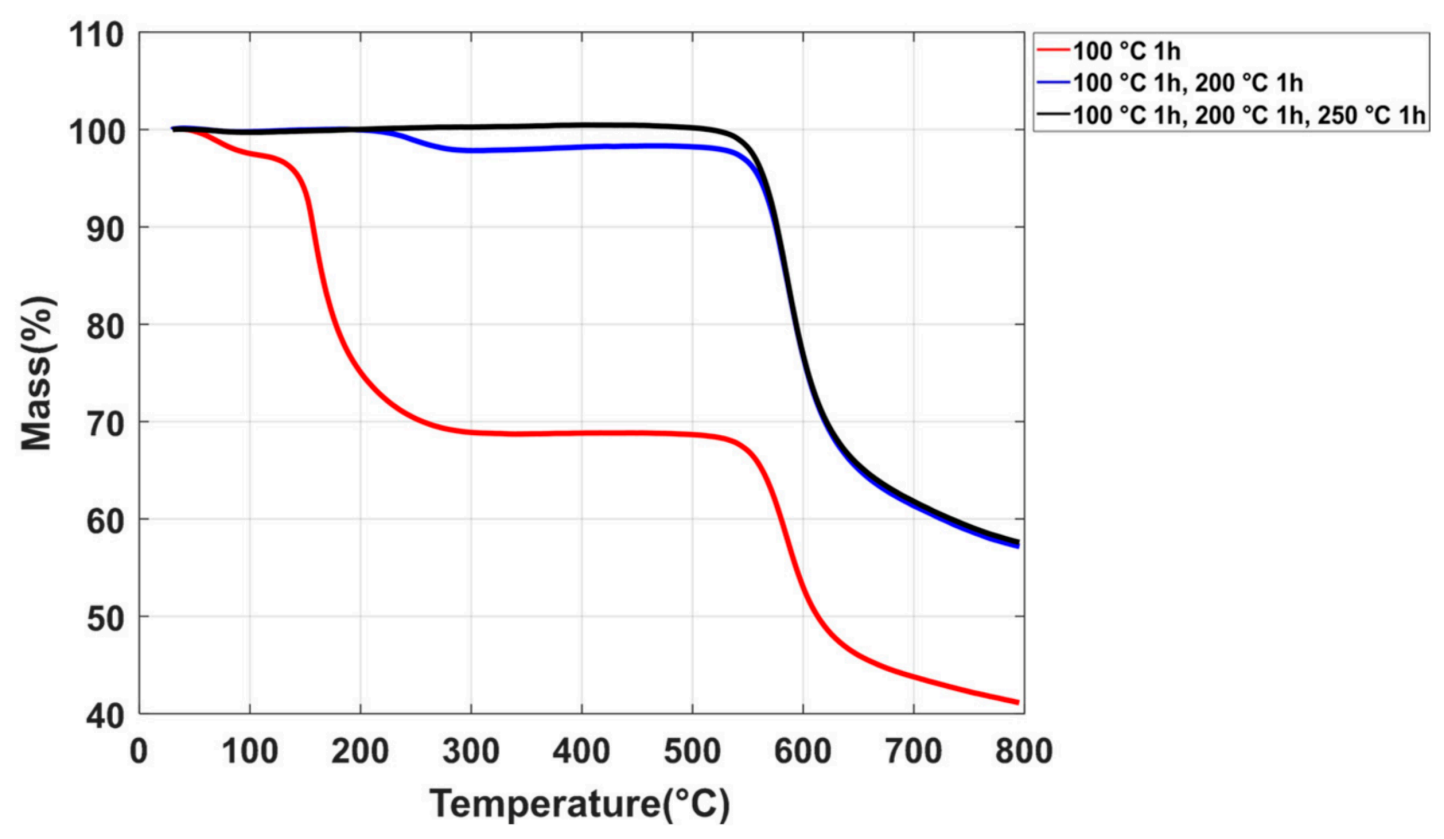
TGA curves of the polyimide films.

Figure 10 shows the TGA results of the PI coating films under different heat treatment conditions. The specimen heat-treated only at 100 °C exhibited a sharp mass loss in the range of approximately 150–200 °C. This indicates that low-temperature heat treatment alone was insufficient to remove DMF, NMP, and the volatile byproducts generated during the imidization process. In particular, the temperature at which the sharp mass loss began is close to the boiling point of DMF, approximately 153 °C. The specimen heat-treated up to 200 °C showed no notable mass loss before 200 °C, but mass loss appeared in the subsequent temperature range. This can be attributed to the removal of DMF during heat treatment, while NMP, with a boiling point of approximately 203 °C, partially remained. The specimen heat-treated up to 250 °C showed no notable mass loss before 500 °C, indicating that the residual solvents were sufficiently removed through heat treatment above the boiling point of NMP. The substantial mass loss occurring above 500 °C is attributed to the thermal decomposition of polyimide, supporting the formation of a thermally stable polyimide structure. Therefore, the TGA results show that the stepwise heat treatment condition up to 250 °C effectively reduced residual volatile substances in the PI coating and confirm the high thermal stability of the PI coating.

### 4.2. Resistance Stability of the Sensor Under Repeated Thermal Cycling

**Figure 11:**
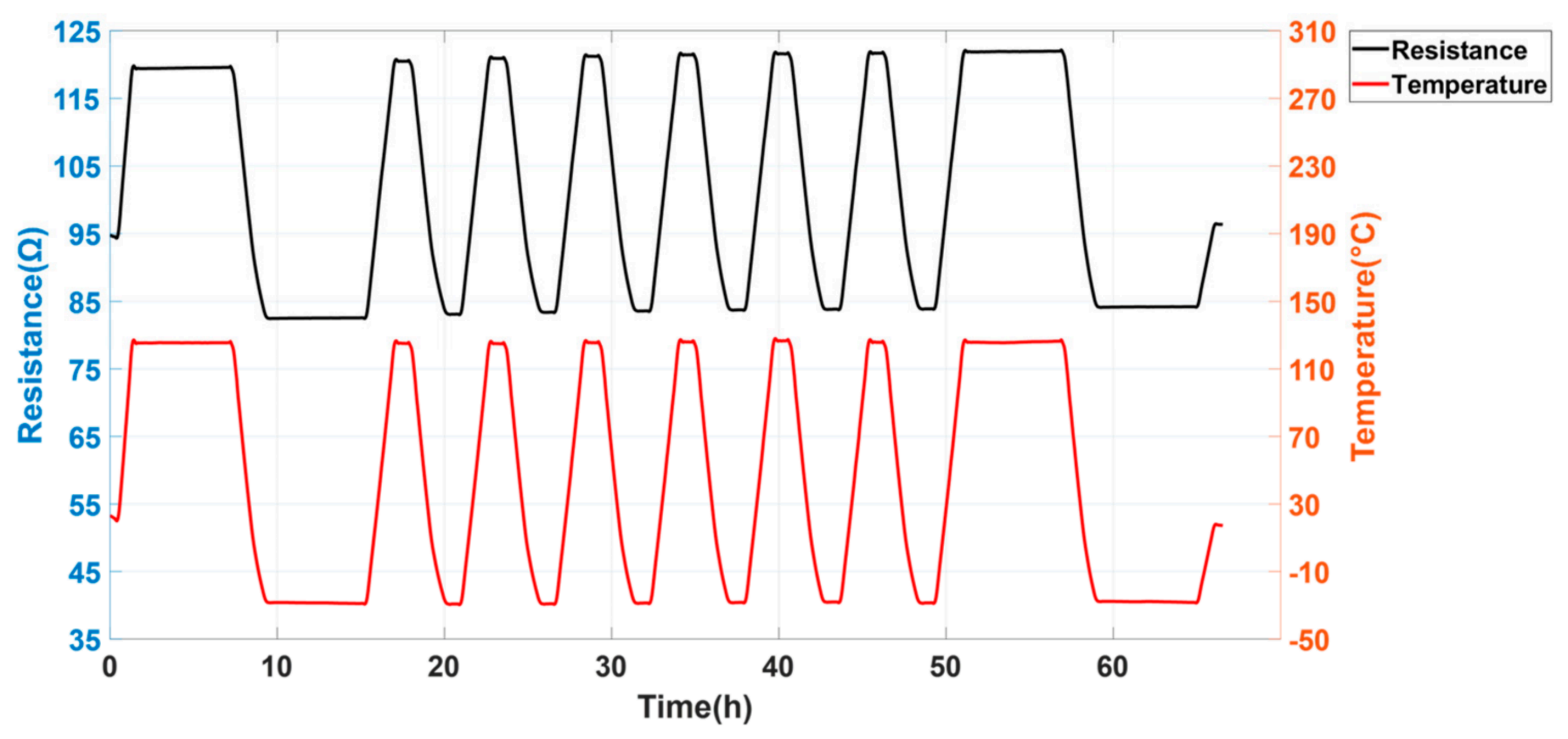
Resistance and temperature curves over eight cycles.

Figure 11 shows the results of the 66-h thermal cycling test comprising eight cycles from −25 °C to 125 °C. The sensor resistance measured in real time and the internal chamber temperature measured by the thermocouple are plotted together on a single graph. The sensor resistance varied linearly with temperature throughout the test, and no abrupt resistance change or noticeable drift was observed. Sensor stability is a critical indicator of RTD performance and can be assessed using the resistance drift rate (DR), as given in Equation (6) [19]. The calculation showed that the sensor resistance at 25 °C was 95.78 Ω at the start of the first cycle and 97.82 Ω at the end of the eighth cycle, yielding a drift rate of 0.032%/h. As a result, the low drift rate maintained over the eight cycles demonstrates the stability of the sensor resistance under repeated thermal cycling. Therefore, this RTD is expected to enable real-time temperature measurement even under repeated high- and low-temperature environments.

(6) $$\mathrm{DR}\;\;=\;\;\frac{\Delta \;R}{R_{0}}\;\;\times \;\;\frac{1}{\Delta \;t}\;\;$$

### 4.3. Calibration Equation for On-Orbit Sensor Data and TCR

**Figure 12:**
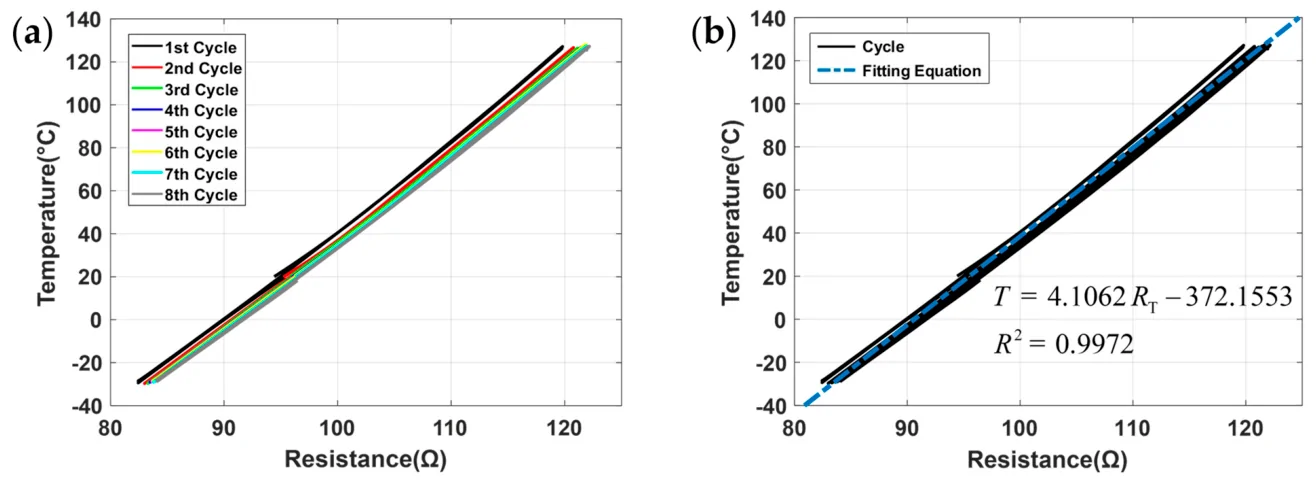
(**a**) Resistance as a function of temperature over eight cycles and (**b**) the same data with the linear fitting result.

Figure 12a shows the temperature-dependent resistance data obtained from the thermal cycling test, with each cycle represented by a different color, whereas Figure 12b presents the cycle-wise data with the linear fitting result. In all cycles, the relationship between temperature and resistance was nearly linear, indicating high linearity. Accordingly, a linear fit was performed on the data using the least-squares method to derive the calibration equation for the fabricated temperature sensor.

*T* = 4.1062*R*_T_ − 372.1553 (7)

The derived calibration equation is given in Equation (7), where *T* is the estimated temperature (°C), and *R*_T_ is the sensor resistance (Ω). This equation is linked to the data processing procedure described in Section 2.2 and will be used to calculate the temperature of the solar panel from ADC values downlinked from orbit. To evaluate the accuracy of the derived calibration equation, the sensor resistance values were substituted into the equation to estimate the corresponding temperatures. The mean absolute error between the estimated and measured temperatures was then calculated and found to be 1.44 °C.

Table 3 compares the temperature coefficient of resistance (TCR) values of Ag-based printed temperature sensors with those reported in previous studies. TCR represents the relative change in resistance per unit change in temperature and is used as an indicator of temperature sensitivity. TCR can be calculated as shown in Equation (8). Using the resistance at 0 °C as the reference, the TCR of the fabricated sensor was calculated to be 0.269%/°C. This value was toward the upper end of the TCR range of 0.077–0.341%/°C reported in previous studies. This indicates that the fabricated sensor exhibited relatively high resistance sensitivity to temperature change.

(8) $$\mathrm{TCR}\;\;[\%/{}^{\circ}C]\;\;=\;\;\frac{1}{R(T_{0})}\;\;\times \frac{R(T)-R(T_{0})}{T-T_{0}}\times \;\;100\;$$

### 4.4. Analysis of Hysteresis Effect on Temperature Estimation

**Figure 13:**
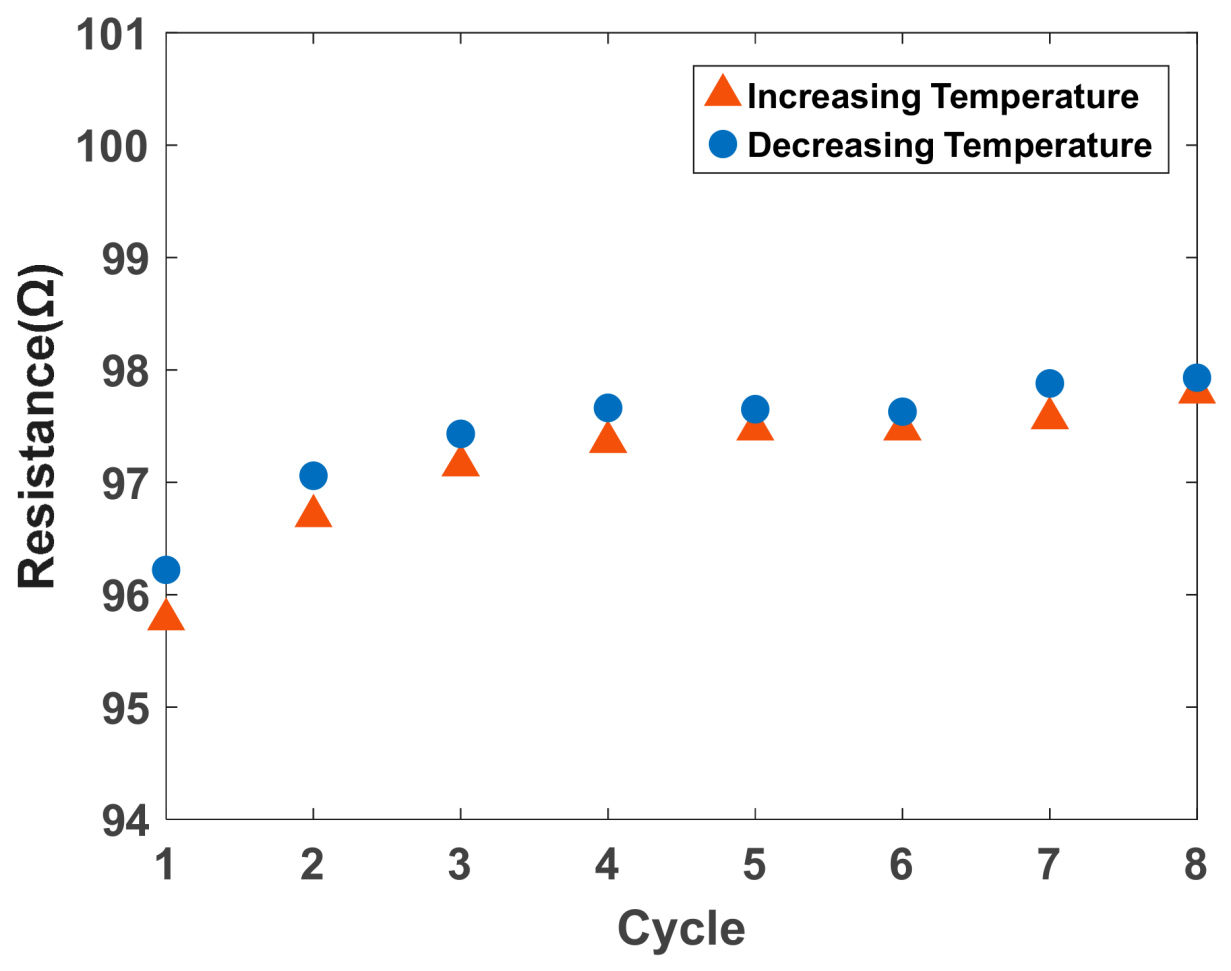
Sensor resistance at 25 °C over eight cycles.

Hysteresis in a temperature sensor refers to the difference in output between the increasing and decreasing temperature segments at the same reference temperature. Greater hysteresis can increase temperature estimation error. To evaluate this, the resistance values obtained during the increasing and decreasing segments of each cycle at 25 °C are plotted in Figure 13 and summarized in Table 4. Using the values recorded in Table 4, the difference between the resistance value in the increasing segment and that in the decreasing segment for each cycle, ∆*R*_Hys_, was calculated. The ∆*R*_Hys_ values for cycles 1–8 were 0.44, 0.35, 0.28, 0.30, 0.17, 0.14, 0.29, and 0.11 Ω, respectively. ∆*R*_Hys_ was largest in the first cycle and generally decreased as the cycles progressed. This behavior is considered to reflect a stabilization process of the sensor during repeated thermal cycling [23]. When the first-cycle ∆*R*_Hys_ value was substituted into the previously derived calibration equation, the resulting temperature deviation between the increasing and decreasing segments was 1.81 °C. The degree of hysteresis can be expressed as a percentage of the full-scale output (FSO). Based on the full temperature range of 150 °C (−25 to 125 °C), the hysteresis was calculated to be 1.2% FSO. This value is similar to the 1.4% FSO reported in a previous study on an Ag-based inkjet-printed temperature sensor characterized according to relevant standards [24]. Therefore, the sensor fabricated in this study exhibits good hysteresis characteristics.

## 5. Conclusions

In this study, an Ag-based flexible RTD was fabricated using an EHD inkjet printing process to measure the surface temperature of INHA RoSAT’s rollable solar panel. The sensor was formed on a flexible PI substrate so as not to interfere with the stowage and deployment of the solar panel. A PI coating was applied to protect the sensor. FT-IR and TGA analyses were performed to evaluate the suitability of the applied PI coating for the space environment. The results confirmed the formation of the imide structure through stepwise heat treatment, a reduction in residual solvents and volatile substances, and thermal stability at high temperatures. A thermal cycling test was performed on the fabricated sensor based on the tailoring concept of MIL-STD-1540. During the test, the sensor resistance varied linearly with temperature and maintained a stable response without abrupt resistance changes or noticeable drift. The drift rate calculated over the entire test was 0.032%/h, and the calibration equation derived from the temperature–resistance data yielded a mean absolute error of 1.44 °C. The TCR was also calculated to be 0.269%/°C, indicating sufficient temperature sensitivity. Hysteresis was calculated to be 1.2% FSO, confirming good hysteresis characteristics even under repeated heating and cooling cycles.

Based on these performance evaluation results, the fabricated flexible temperature sensor was attached to the surface of INHA RoSAT’s rollable solar panel, integrated into the spacecraft, and launched into orbit. This study covered the sensor development process, including sensor design, assessment of process suitability, and performance evaluation under thermal cycling. Once on-orbit data become available, the calibration equation derived in this study is expected to be used to estimate the solar panel’s surface temperature. The estimated temperature data can then be used to analyze the effect of temperature variation on power generation performance and support satellite power management.

## Figures and Tables

**Table 1:** Conditions of EHD inkjet printing.

Print Speed [mm/s]	Working Position [μm]	Voltage [kV]	Flow Rate [μL/min]
100	1800	2	5.5

**Table 2:** Specifications of the fabricated sensor.

Sensing Material/ Substrate	Line Length (mm)	Average Line Width (μm)	Sensor Thickness (μm)	Initial Resistance (Ω, at 25 °C)
Ag/PI	50	200	60	95

**Table 3:** Comparison of TCR values of printed temperature sensors.

Reference	Material/Substrate	Printing Method	T Range (°C)	TCR Value (%/°C)
[20]	Ag/PI	Inkjet	[20, 60]	0.223
[21]	AgNP/PI	Inkjet	[−50, −10]	0.269
[22]	AgNP/PET	EHD Inkjet	[20, 120]	0.077
[11]	AgNP/PET	EHD Inkjet	[20, 110]	0.341
This work	Ag/PI	EHD Inkjet	[−25, 125]	0.269

**Table 4:** Resistance values corresponding to Figure 13.

Cycle	1st	2nd	3rd	4th	5th	6th	7th	8th
Increasing (Ω)	95.78	96.70	97.15	97.36	97.48	97.48	97.58	97.82
Decreasing (Ω)	96.22	97.05	97.43	97.66	97.65	97.62	97.87	97.93
∆*R*_Hys_ (Ω)	0.44	0.35	0.28	0.30	0.17	0.14	0.29	0.11

## Data Availability

The data presented in this study are available from the corresponding author upon reasonable request.
